# Nuocytes from mesenteric lymph node promote allergic responses in a mouse model

**DOI:** 10.1016/j.bjorl.2019.12.010

**Published:** 2020-02-12

**Authors:** Lin Lin, Xinyue Tang, Zheng Chen, Jinjin Wei, Fei Dai, Guangbin Sun

**Affiliations:** a Huashan Hospital of Fudan University, Department of Otorhinolaryngology-Head and Neck Surgery, Shanghai, China; b Huashan Hospital North of Fudan University, Department of Otorhinolaryngology-Head and Neck Surgery, Shanghai, China

**Keywords:** Lymphoid cells, Lymph node, Allergic rhinitis, Mice, Animal models

## Abstract

**Introduction:**

Nuocytes play an important role in Type 2 immunity. However, the contribution of ILC2s to allergic rhinitis remains to be clearly elucidated.

**Objective:**

To evaluate the role of nuocytes from mesenteric lymph node on allergic responses in mice.

**Methods:**

After intraperitoneal administration of interleukin IL-25 and IL-33 to wild-type and *Il17br^−/−^Il1rl1^−/−^* double-deficient mice, nuocytes were purified from the the nasal-associated lymphoid tissue and mesenteric lymph nodes. Then, we assessed productions of IL-5 and IL-13 in nuocytes’ cultures. Finally, we adoptively transferred the mesenteric lymph node-derived nuocytes from wild-type and *Il17br^−/−^Il1rl1^−/−^* mice to the murine model of allergic rhinitis to evaluate their roles in nasal allergic responses.

**Results:**

We showed that nuocytes in the mesenteric lymph nodes of wild-type mice were upregulated after application of IL-25 and IL-33, and were induced to produce IL-5 and IL-13. Numbers of sneezing and nasal rubbing as well as eosinophils were all enhanced after the adoptive transfer of wild-type nuocytes. Concentrations of IL-5, IL-13, IL-25 and IL-33 in nasal lavage fluid of allergic mice were also increased. However, nuocytes from*Il17br^−/−^Il1rl1^−/−^* mice did not increase sneezing and nasal rubbing and eosinophilia, and upregulate the above cytokines in the nasal lavage fluid.

**Conclusion:**

The findings demonstrate that nuocytes from the mesenteric lymph nodes of wild-type mice promote allergic responses in a mouse model.

## Introduction

Allergic rhinitis (AR) is an immunoglobulin E (IgE)-mediated immune disorder induced by allergen exposure, which is characterized by a type 2 helper T (Th2) polarized inflammation. Th2 cytokines like Interleukin (IL)-5 and IL-13 are the essential pathogenic factors in the initiation and development of allergic condition.^1^ IL-5 accelerates nasal mucosa eosinophilia, and upregulates the number of activated eosinophils. IL-13 regulates airway hyper-responsiveness, mucus cell metaplasia and tissue fibrosis.^1^

Although Th2 cell is an essential source of Type 2 cytokines in adaptive immune response, many studies have shown that innate lymphoid cell (ILC) provides another source of this type cytokines, such as IL-5 and IL-13.^2^ ILC populations have been categorized into three groups according to a proposed nomenclature.^3^ Although differences among these cell types exist, they have in common productions of IL-5 and IL-13 in response to the IL-17 family member IL-25 (IL-17E) and the IL-1 family member IL-33 produced from the airway epithelial cells.^3^

Nuocytes belong to type 2 ILCs (ILC2s), which are identified as lineage (lymphocyte, macrophage, dendritic cell, basophil, eosinophil, mast cell, and natural killer cell) negative and inducible costimulator (ICOS) positive.^4^ These cells express both the IL-25 receptor (IL-17BR) and the IL-33 receptor (IL1RL1 or T1/ST2), and expand in vivo in response to the Type 2-inducing cytokines IL-25 and IL-33.^4^ Nuocytes represent the predominant early source of IL-13 during helminth infection. In the combined absence of IL-25 and IL-33 signaling, nuocytes fail to expand, resulting in a severe defect in worm expulsion that is rescued by the adoptive transfer of in vitro cultured wild-type nuocytes.^4^ In spite of proliferation by these two cytokines, nuocytes are induced to secrete IL-5 and IL-13 but little IL-4 by both IL-25 and IL-33 during *Nippostrongylus brasiliensis* infection in the gut. Besides parasites expulsion, innate IL-13-producing nuocytes arise during allergic lung inflammation and contribute to airways hyperreactivity.^5^

A previous research study from our laboratory showed that nuocytes could be induced by ovalbumin (OVA) in the nasal-associated lymphoid tissue (NALT) of the murine AR model, and could produce IL-5 and IL-13 after the administration of recombinant (rm) IL-25.^6^ That study indicated that nuocytes might play a proinflammatory role in the allergic state of mice models.^6^ Another of our studies evaluated the expression of Orai1 in nuocytes, the key component of Ca^2+^ release-activated Ca^2+^ channel in plasma membrane of various cells,^7^ and the function of this protein in nuocytes (unpublished data). In the present study, we aimed to evaluate the role of nuocytes from mesenteric lymph node (mLN) on allergic responses in mice.

## Methods

### Mice

Female BALB/c mice (6–8 weeks old) were purchased from the Chinese Academy of Sciences Shanghai Laboratory Animal Center. *Il17br^−/−^Il1rl1^−/−^* double-deficient mice on a BALB/c background (6–8 weeks old) were purchased from Cyagen US Inc. Santa Clara, CA, USA. The mice were maintained in horizontal laminar flow cabinets and provided sterile food and water in a specific pathogen-free facility. The animal studies were approved by the Institutional Animal Care and Use Committee of Fudan University (ethic number: 201808001Z). These mice were randomly divided into five groups (n = 12 for each group).

### Nasal allergic animal models

**Figure 1:**
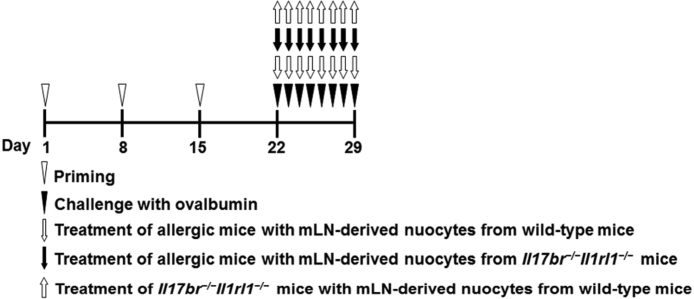
Study protocols.

According to the published procedures,^8^ these mice were administered 0.5 mg/mL of OVA (grade V; Sigma-Aldrich, St. Louis, Missouri) and 20 mg/mL of aluminium hydroxide (Alum) (Sinopharm Chemical Reagent Co Ltd., Shanghai, China) in normal saline at a dosage of 0.2 mL per mouse by intraperitoneal injection. The sensitization was repeated three times at weekly intervals (days 1, 8 and 15). Then, the mice were challenged by daily instillation of OVA solution droplet (40 mg/mL in normal saline) into the nostrils (0.02 mL per mouse) with a micropipette on days 22–29 (Fig. 1). As a negative control, a group of mice received the challenge treatment of normal saline alone (normal group). Two groups of allergic mice (wild group: treatment of allergic mice with nuocytes from wild-type mice; *Il17br^−/−^Il1rl1^−/−^* group: treatment of allergic mice with nuocytes from *Il17br^−/−^Il1rl1^−/−^* mice) received the adoptive transfer of nuocytes from wild-type mice or *Il17br^−/−^Il1rl1^−/−^* mice cultured in vitro intravenously in the tail vein on challenging days, respectively, whereas another group of allergic mice was not treated with nuocytes (AR group). In addition, another group of *Il17br^−/−^Il1rl1^−/−^*mice received the adoptive transfer of nuocytes from wild-type mice cultured in vitro intravenously in the tail vein on challenging days. Relevant nasal symptoms were evaluated by counting numbers of sneezes and nasal rubs during 10 min immediately after the last OVA intranasal provocation on Day 29.

### NALT and mLN cells preparation

0.4 μg per dose of mouse rmIL-25 (R&D Systems, Inc., Minneapolis, MN, USA) and rmIL-33 (R&D Systems, Inc., Minneapolis, MN, USA) in Phosphate-Buffered Saline (PBS) was administered daily for 3 d intraperitoneally. Control animals received PBS only. Mice were sacrificed 24 h later, and the foreteeth were cut off. The lower jaw and cheek muscles were removed, and NALT was exposed by carefully peeling away the plate. NALT was localized on the posterior part of the plate, and teased out with syringe needles in ice-cold RPMI 1640 culture medium supplemented with 10% FBS. mLN tissues were also obtained. All these tissues were minced using sterile scissors to approximately <2 mm pieces, and digested through 200 μL of 200 U/Ml collagenase Type III (Sigma–Aldrich, St. Louis, MO, USA) and 200 μL of 200 μg/mL DNase Type IV (Sigma–Aldrich, St. Louis, MO, USA), and then passed through a 70 μm Falcon cell strainer. RBC were lysed in Tris-buffered ammonium chloride solution (0.83% NH4Cl and 20 mM Tris/Cl).

### Flow cytometry analysis

NALT and mLN cell suspensions from wild-type and *Il17br^−/−^Il1rl1^−/−^* mice were centrifuged for 10 min at 200×*g* at 4 °C, and cells were harvested for flow cytometry analysis. These cells were washed with 200 μL flow cytometry medium [FCM, 1% BSA and 0.1% sodium azide in PBS] and incubated with 50 μL rabbit serum for 5 min at room temperature to block the Fcγ receptor. These cells then were washed three times with FCM, and stained with 50 μL of monoclonal antibodies against the following surface molecules at 4 °C for 30 min at 1 × 10^8^ cells/mL: CD3, CD4, CD8, CD11b, CD11c, CD19, FcεR1, IL17BR; all antibodies purchased from MyBioSource, Inc., San Diego, CA, USA or BioLegend, San Diego, CA, USA and conjugated to fluorescein isothiocyanate and ICOS monoclonal antibody (purchased from MyBioSource, Inc., San Diego, CA and conjugated to Phycoerythrin). They were washed again and incubated with 50 μL secondary antibody (biotin-conjugated rabbit anti-mouse IgG; Dako Denmark A/S, Glostrup, Denmark) followed by 50 μL R-phycoerythrin-conjugated streptavidin (RPE-streptavidin; Dako Denmark A/S, Glostrup, Denmark). After staining, the cells were resuspended in staining buffer containing 4′, 6-Diamidino-2-Phenylindole Dihydrochloride (DAPI) for the exclusion of dead cells. Controls for single color analysis were carried out by incubating cells with a primary antibody of the same isotype, but of irrelevant specificity at matched concentrations followed by secondary reagents, as described above. The samples were collected on an LSR II flow cytometer equipped with FACSDIVA software (BD Biosciences, Mountain View, CA, USA) and analyzed with FlowJo version 10 (Tree Star, Inc., Ashland, OR). Set-up of the instrument was carried out visually, and selection of the isotype control antibody was in accordance with the primary antibodies’ host species, isotype, and conjugation format.

### Nuocytes cultures

Nuocytes from NALT and mLN of wild-type and *Il17br^−/−^Il1rl1^−/−^*mice were all sorted and purified as CD3CD4CD8CD19CD11bCD11cFcεR1 (lineage)^−^ICOS^+^ cells at a maximum concentration of 6 × 10^7^ cells/mL. They were cultured for 6d in RPMI containing 10% FCS, 1% penicillin/streptomycin, 0.1% β-mercaptoethanol, 10 ng/mL of mouse rmIL-7 (BioLegend, San Diego, CA, USA), and 10 ng/mL of mouse rmIL-33 (BioLegend, San Diego, CA, USA).

Then, the cells from mLN of wild-type and *Il17br^−/−^Il1rl1^−/−^* mice were incubated with 100 ng/mL of rmIL-25 (BioLegend, San Diego, CA, USA) and 100 ng/mL of rmIL-33 (BioLegend, San Diego, CA, USA) in the cultures. The cultures were kept for 3d. The cell supernatants were used to measure IL-5 and IL-13 production by Enzyme-Linked Immunosorbent Assay (ELISA), and the cells were used to assess contents of messenger (m) RNAs of IL-5 and IL-13 by Real-Time reverse Transcription-Polymerase Chain Reaction (RT-PCR). After that, nuocytes from wild-type and *Il17br^−/−^Il1rl1^−/−^*mice or nuocytes from wild-type mice were resuspended in normal saline, and were adoptively transferred into AR or *Il17br^−/−^Il1rl1^−/−^* mice at 100 µL per mouse intravenously in the tail vein on challenging days.

### Immunocytochemistry and confocal microscopy

Nuocytes from mLN of wild-type and *Il17br^−/−^Il1rl1^−/−^* mice were seeded on glass coverslips and grown for 48 h. Adhering cells were washed twice with PBS and fixed with 4% paraformaldehyde for 5 min at room temperature, then, rinsed three times with PBS containing 0.2% Triton X-100 (PBS-T), and blocked with 3% bovine serum albumin (in PBS) for 30 min. Samples were then incubated overnight at room temperature with primary antibodies against IL-13. After rinsing with PBS (3 × 5 min), samples were incubated 1 h at room temperature with fluorescein isothiocyanate–conjugated secondary antibodies in the darkness. Coverslips were then washed twice with PBS-T before nucleus staining with 10 mg/mL DAPI for 5 min at room temperature. Finally, the coverslips were washed three times by PBS-T and twice by PBS before mounting with Vectashield (Vector Laboratories, Burlingame, CA, USA). As negative controls, cell samples were incubated either with the secondary antibody alone or with the primary antibody preadsorbed with a specific blocking peptide (1:5 w/w). Images were obtained with a Leica TCS SP5 Confocal Laser Scanning Microscope by the Leica Confocal Software (LCS) 2.61 (Leica Microsystems, Wetzlar, Germany). Images were analyzed by LCS Lite (Leica) software. To enable comparison, all images were acquired using the same parameters of laser power and photomultiplier sensitivity. Images shown are representative of at least three separate experiments in each condition, and were processed with identical values for contrast and brightness.

### Nasal lavage fluid (NLF)

After sacrifice of the mice, one blunted 18 gauge needle was pointed toward the heads of a portion of mice for NLF. One injection of 2000 μL normal saline was performed and the fluid was collected with a tube under both nares of the nose for NLF. A portion of NLF was centrifuged for 10 min at 150×*g* at 4 °C. The supernatants were stored at −70 °C for IL-5 and IL-13 assays. Another portion of NLF was collected for detection of eosinophils, and differential cell counts on 150 cells were performed on cytospins (Cytospin 4 Shandon Ltd., Runcorn, UK) stained with Giemsa.^9^

### ELISA analysis

IL-5 in the culture and the NLF was measured using a 96 well microplate coated with 1 μg/mL of IL-5 rabbit antibody. The microplate was incubated in 3% bovine serum albumin at 37 °C for 1 h. Samples at 1:10 dilution were then added followed by incubation at 37 °C for 1 h. After washing, anti-IL-5 antibody (R&D Systems, Inc., Minneapolis, MN, USA) that had been biotinylated using a biotinylation kit (American Qualex International Inc, San Clemente, CA, USA) was then added at 1 μg/mL and allowed to incubate at 25 °C for 1 h. After washing, 1.5 μg/mL of streptavidin peroxidase was added followed by incubation at 25 °C for 1 h. After washing, Tetramethylbenzidine (TMB) substrate (12.5 mL of citric-phosphate buffer, 200 μL of TMB stock solution [6 mg/mL in dimethyl sulfoxide], 100 μL of 1% hydrogen peroxide; FlukaChemical Co, Ronkonkoma, NY, USA) was added to produce a color reaction. The reaction was terminated by the addition of 6N sulfuric acid. Optical density was determined at 450 nm using a microplate reader (Microplate Reader MTP-32; Corona Electric, Ibaraki, Japan). Concentrations of IL-13 in the culture, and IL-13, IL-25 and IL-33 in the NLF were evaluated using corresponding ELISA kits (purchased from R&D Systems, Inc., Minneapolis, MN, USA or MyBioSource, Inc. San Diego, CA, USA).

### Real-time RT-PCR analysis

mRNAs of IL-5 and IL-13 in the nuocytes cultures were analyzed by real-time RT-PCR. Total RNA of a portion of samples was extracted with Trizol (Invitrogen, Carlsbad, CA, USA) and treated with RNase-free DNase. For reverse transcription, 2 μg of above RNA was reversely transcribed with random hexamers (Invitrogen, Carlsbad, CA, USA) and cDNA was amplified according to the manufacturer’s instructions. Primers were designed using Primer Express software (ABI) from sequence available in Genbank and were synthesized (Geneland Biotech, Shanghai, China). Real-time RT-PCR was performed to detect mRNAs of IL-5 and IL-13. IL-5 primers were: forward primer 5′-TCACCGAGCTCTGTTGACAA-3′ and reverse primer 5′-CCACACTTCTCTTTTTGGCG-3′. IL-13 primers were: forward primer 5′-AGACCAGACTCCCCTGTGCA-3′ and reverse primer 5′-TGGGTCCTGTAGATGGCATTG-3′. GAPDH mRNA was also examined to control the sample-to-sample variation in RNA isolation and integrity by using a pair of primers: forward primer 5′-ACCACAGTCCATGCCATCAC-3′ and reverse primer 5′-TCCACCACCCTGTTGCTGTA-3′. After initial denaturation at 95 °C for 10 min, the amplification profile was 15 s of denaturation at 95 °C, 1 min of annealing and extension at 60 °C for 45 cycles. Negative control RT reaction mixtures contained no reverse transcriptase and no cDNA in the PCR amplification mixtures. For measurement 2 μL of diluted cDNA was amplified in a total reaction volume of 20 μL by using an 7500 real-time PCR System (Applied Biosystems, Foster City, CA, USA) with 20 × SYBR Green mixture (Invitrogen, Carlsbad, CA, USA). Specificity of PCR products was evaluated by melting curve analysis and by size in agarose gels. Using three dilutions of cDNA, linearity of PCR amplification was controlled. Evaluation of data was performed using the ΔCT method with GAPDH as internal standard.

### Statistical analysis

Statistical analysis was performed with a commercially available statistical software prism 6.0 (GraphPad Software Inc., San Diego, CA, USA). Data were analyzed using the unpaired Student *t* test ± Welch’s correction and presented as mean ± SEM (standard error of the mean). Significance of a difference was accepted at the 5% level of confidence; *p* < 0.05 was considered statistically significant.

## Results

### Nuocytes in the NALT of mice

**Figure 2:**
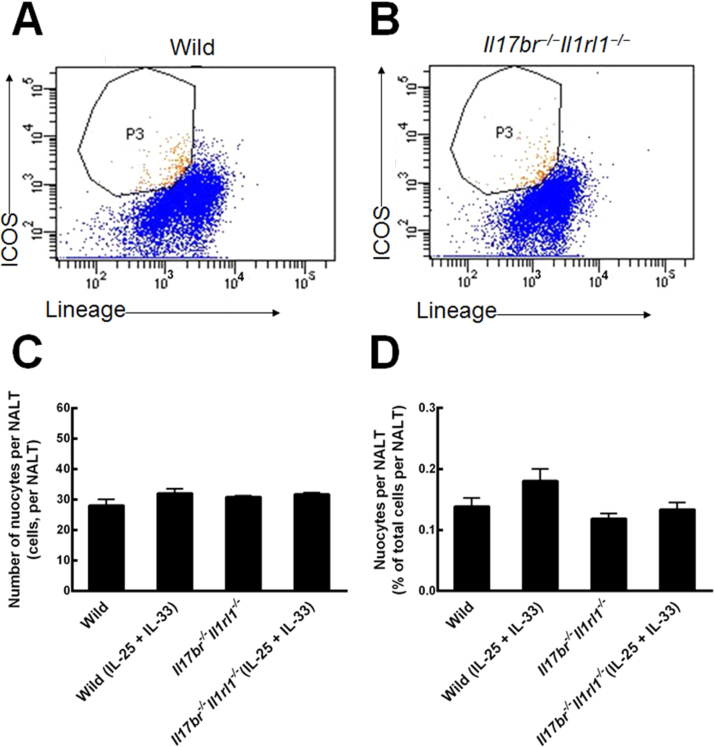
Nuocytes from NALT. A, Nuocytes from wild-type mice. B, Nuocytes from *Il17br^−/−^Il1rl1^−/−^* mice. C, Number of nuocytes. D, Percentage of nuocytes. Each value represents the mean (SEM) of 6 mice in each group. Wild, wild-type mice; Wild (IL-25+IL-33), wild-type mice with the treatment of rmIL-25+rmIL-33; *Il17br^−/−^Il1rl1^−/−^*, *Il17br^−/−^Il1rl1^−/−^*mice; *Il17br^−/−^Il1rl1^−/−^* (IL-25+IL-33), *Il17br^−/−^Il1rl1^−/−^* mice with the treatment of rmIL-25+rmIL-33.

Nuocytes from the NALT of wild-type and *Il17br^−/−^Il1rl1^−/−^* mice were sorted and purified as lineage^−^ICOS^+^ cells (Fig. 2 A and B).^6^ Comparisons of wild-type nuocytes number and percentage indicated that their total number and percentage were not enhanced statistically in the NALT in response to intraperitoneal rmIL-25 and rmIL-33 treatment (Fig. 2 C and D). As for *Il17br^−/−^Il1rl1^−/−^* mice, the number and percentage of nuocytes also showed no significant increase after rmIL-25 and rmIL-33 administration (Fig. 2C and D). Furthermore, there were no statistical differences between wild-type and *Il17br^−/−^Il1rl1^−/−^* nuocytes in the number and percentage per NALT before and after rmIL-25 and rmIL-33 treatment (Fig. 2 C and D). From the results, we conclude intraperitoneal administration of rmIL-25 and rmIL-33 cannot influence proliferation of nuocytes located to NALT.

### Nuocytes in the mLN of mice

**Figure 3:**
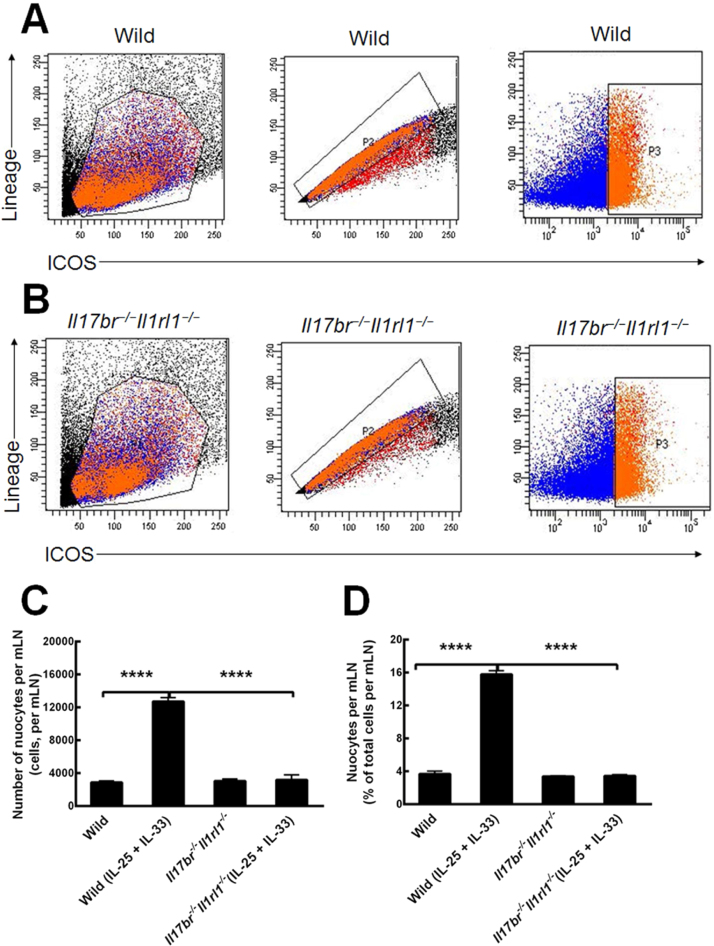
Nuocytes from mesenteric lymph node (mLN). A, Nuocytes from wild-type mice. B, Nuocytes from *Il17br^−/−^Il1rl1^−/−^* mice. C, Number of nuocytes per mLN. D, Percentage of nuocytes per mLN. Each value represents the mean (SEM) of 6 mice in each group. Wild, wild-type mice; Wild (IL-25+IL-33), wild-type mice with the treatment of rmIL-25+rmIL-33; *Il17br^−/−^Il1rl1^−/−^*, *Il17br^−/−^Il1rl1^−/−^*mice; *Il17br^−/−^Il1rl1^−/−^* (IL-25+IL-33), *Il17br^−/−^Il1rl1^−/−^*mice with the treatment of rmIL-25+rmIL-33. ****p < 0.0001 vs. Wild (IL-25+IL-33).

Nuocytes from mLN of wild-type and *Il17br^−/−^Il1rl1^−/−^*mice were also sorted and purified as lineage^−^ICOS^+^ cells (Fig. 3 A and B).^4^ Comparisons of wild-type nuocytes number and percentage demonstrated that the total number and percentage were enhanced statistically in the mLN in response to intraperitoneal rmIL-25 and rmIL-33 treatment (Fig. 3 C and D). In the way of *Il17br^−/−^Il1rl1^−/−^* mice, nuocytes number and percentage indicated no significant increase after rmIL-25 and rmIL-33 application (Fig. 3 C and D). In addition, there were no statistical differences between wild-type and *Il17br^−/−^Il1rl1^−/−^*nuocytes in the number and percentage per mLN before the cytokines treatment, and big differences after the cytokines treatment (Fig. 3 C and D). The data demonstrate that nuocytes fail to expand in the combined *Il17br^−/−^Il1rl1^−/−^* mice in the mLN in response to rmIL-25 and rmIL-33,^4^ and IL-25 and IL-33 are required for nuocytes generation during the type-2 inflammation.

### IL-5 and IL-13 expressions in mLN-derived nuocytes cultures after rmIL-25 and rmIL-33 administration

**Figure 4:**
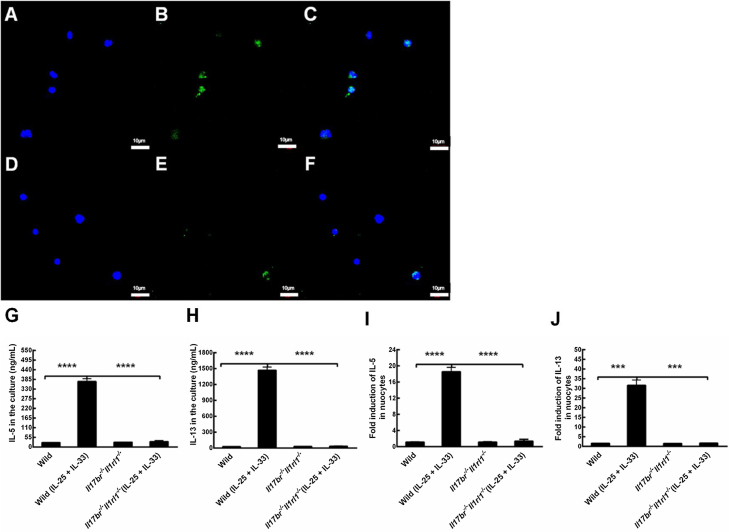
Expression of IL-13 in nuocytes, and responses of mesenteric Lymph Node (mLN)-derived nuocytes to mouse recombinant (rm) Interleukin (IL)-25 and IL-33 in the culture. A, IL-13 in wild-type mice [4′, 6-Diamidino-2-phenylindole dihydrochloride (DAPI)]. B, IL-13 in wild-type mice (fluorescein isothiocyanate). C, IL-13 in wild-type mice (merge). D, IL-13 in *Il17br^−/−^Il1rl1^−/−^* mice (DAPI). E, IL-13 in *Il17br^−/−^Il1rl1^−/−^* mice (fluorescein isothiocyanate). F, IL-13 in *Il17br^−/−^Il1rl1^−/−^* mice (merge). G, IL-5 in the culture. H, IL-13 in the culture. I, IL-5 mRNA in nuocytes. J, IL-13 mRNA in nuocytes. Scale bars: 10 μm. Original magnification: ×400. Each value represents the mean (SEM) of 6 mice in each group. Wild, wild-type mice; Wild (IL-25+IL-33), wild-type mice with the treatment of rmIL-25+rmIL-33; *Il17br^−/−^Il1rl1^−/−^*, *Il17br^−/−^Il1rl1^−/−^*mice; *Il17br^−/−^Il1rl1^−/−^* (IL-25+IL-33), *Il17br^−/−^Il1rl1^−/−^* mice with the treatment of rmIL-25+rmIL-33. ****p < 0.0001 vs. Wild (IL-25 + IL-33). ***p < 0.001 vs. Wild (IL-25+IL-33).

To investigate whether the mLN-derived nuocytes produce Type 2 cytokines in wild-type and *Il17br^−/−^Il1rl1^−/−^*mice, we determined IL-13 expression using immunocytochemistry and confocal microscopy, and evaluated contents of IL-5 and IL-13 in responses to rmIL-25 and rmIL-33 in the culture using ELISA and real-time RT-PCR. IL-13 was expressed in wild-type and *Il17br^−/−^Il1rl1^−/−^* nuocytes according to immunofluorescence analysis (Fig. 4 A–F). There were no statistical differences in proteins and mRNAs of IL-5 and IL-13 between these two type nuocytes before rmIL-25 and rmIL-33 treatment (Fig. 4 G–J). Expressions of IL-5 and IL-13 were augmented significantly after these two cytokines administration in wild-type nuocytes whether in protein and mRNA, in the meantime; there were significant differences between these two type nuocytes in expressions of these Type 2 cytokines (Fig. 4 G–J). However, IL-5 and IL-13 concentrations were not upregulated in *Il17br^−/−^Il1rl1^−/−^* nuocytes after the above treatment (Fig. 4 G, H, I and J). The findings indicate that combined *Il17br^−/−^Il1rl1^−/−^* knockout in mice deprives nuocytes of responses to IL-25 and IL-33, which contributes to no upregulation of IL-5 and IL-13.^4^

### Aggravation of allergic responses in mice models by wild-type nuocytes from the mLN

**Figure 5:**
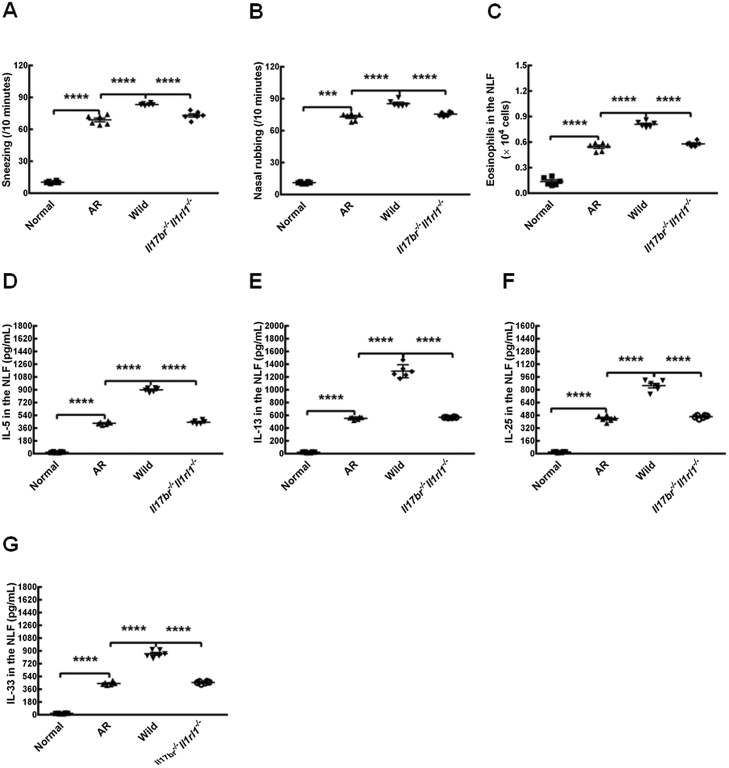
Aggravation of allergic inflammation in mice models by wild-type nuocytes from mesenteric Lymph Node (mLN). A, Sneezing. B, Nasal rubbing. C, Eosinophils in the Nasal Lavage Fluid (NLF). D, Interleukin (IL)-5 in the NLF. E, IL-13 in the NLF. F, IL-25 in the NLF. G, IL-33 in the NLF. Each value represents the mean (SEM) of 6 mice in each group. Normal, normal group; AR, AR group; Wild, treatment of allergic mice with mLN-derived nuocytes from wild-type mice; Il17br^−/−^Il1rl1^−/−^, treatment of allergic mice with mLN-derived nuocytes from Il17br^−/−^Il1rl1^−/−^ mice. ****p < 0.0001 vs. AR or Wild. ***p < 0.001 vs. AR.

To evaluate allergic reactions by OVA-established wild-type mice models, sneezing and nasal rubbing were counted after the last challenge. AR mice showed statistically increased numbers of sneezing and nasal rubbing compared with those of normal mice (Fig. 5 A and B). Numbers of eosinophils distributed in the NLF, and contents of IL-5, IL-13, IL-25 and IL-33 in the NLF of AR mice also displayed enhancements compared to those in normal mice (Fig. 5 C). The results clearly suggest that the murine model of AR was settled successfully. The adoptive transfer of nuocytes from wild-type mice mLN intravenously in the tail vein augmented numbers of sneezing, nasal rubbing and eosinophils, and concentrations of IL-5, IL-13, IL-25 and IL-33 in the NLF compared to those in AR mice (Fig. 5 A–G). Additionally, there were statistical differences between treatments with wild-type nuocytes and with *Il17br^−/−^Il1rl1^−/−^* nuocytes whether in numbers of sneezes, nasal rubs and eosinophils, or in levels of Type 2 cytokines in the NLF (Fig. 5 A–G). However, all of the above parameters were unchanged statistically after the adoptive transfer of *Il17br^−/−^Il1rl1^−/−^* nuocytes into AR mice (Fig. 5 A–G). These findings demonstrate that nuocytes aggravate allergic reponse in AR mice possibly through the upregulated expressions of IL-5 and IL-13 by IL-25 and IL-33 produced from nasal mucosa epithelium,^10^ and the further increased productions of IL-25 and IL-33 by epithelial cells after the stimulation of nuocytes.

### Restoration of allergic responses in mice models after the adoptive transfer of wild-type mLN-derived nuocytes into the combined Il17br^−/−^Il1rl1^−/−^ mice

**Figure 6:**
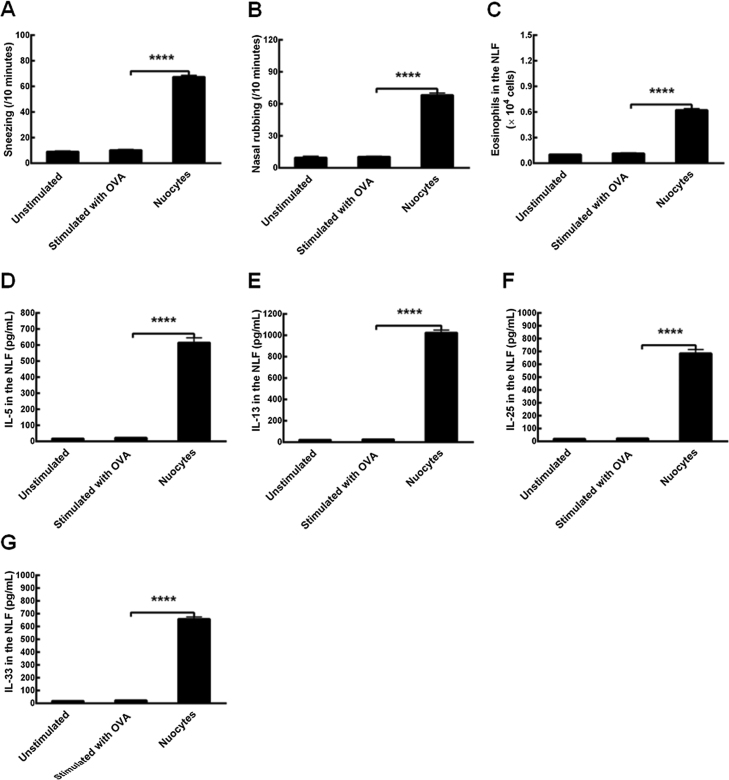
Restoration of allergic inflammation in combined *Il17br^−/−^Il1rl1^−/−^* mice models. A, Sneezing. B, Nasal rubbing. C, Eosinophils in the Nasal Lavage Fluid (NLF). D, Interleukin (IL)-5 in the NLF. E, IL-13 in the NLF. F, IL-25 in the NLF. G, IL-33 in the NLF. Each value represents the mean (SEM) of 6 mice in each group. Unstimulated, treatment without Ovalbumin (OVA); Stimulated with OVA, treatment with OVA; Nuocytes, treatment of *Il17br^−/−^Il1rl1^−/−^* mice with mLN-derived nuocytes from wild-type mice. ****p < 0.0001 vs. Stimulated with OVA.

To determine whether OVA could establish AR models in *Il17br^−/−^Il1rl1^−/−^*mice, we carried out the protocols with the allergen and alum according to the same procedures.^8^ Strikingly, there were no significant differences in numbers of sneezes, nasal rubs and eosinophils, or in levels of Type 2 cytokines in the NLF between unstimulated (treatment without OVA) and stimulated *Il17br^−/−^Il1rl1^−/−^* mice with OVA (treatment with OVA) (Fig. 6 A–G). However, after the adoptive transfer of wild-type nuocytes into the combined *Il17br^−/−^Il1rl1^−/−^*mice, the latter upregulated above parameters statistically (Fig. 6 A–G). In the meantime, mice models of AR were established successfully, and the allergic responses was restored. The results suggest that ILC2s and innate Type 2 cytokines are indispensable for the early stage of allergic condition.

## Discussion

Recent advances in our understanding of proallergic cytokines and ILC2s indicate their essential roles in Type 2 immunity-mediated disorders such as allergic nasal diseases, asthma and atopic dermatitis.^11^ Proallergic cytokines like IL-25, IL-33 and thymic stromal lymphopoietin are released from epithelial cells in inflamed tissues, and drive allergic inflammation by acting on innate and adaptive immune systems.^12^ ILC2s, such as nuocytes,^4^ natural helper cells^13^ and innate Type 2 helper cells,^14^ are an innate immune population that responds to proallergic cytokines IL-25 and IL-33 by producing Type 2 cytokines.

Studies show that the cytokines IL-25 and IL-33 are important initiators of Type 2 inflammation. Treatment of mice with IL-25 or IL-33 provokes strong induction of IL-5, IL-13, and Type 2 pathology.^15^, ^16^ IL-25 and IL-33 double-deficient mice also display a delayed or reduced antigen-specific Type 2 response.^17^, ^18^ Additionally, although IL-25 and IL-33 belong to different cytokine families, IL-17 and IL-1, respectively, they result in similar reactions.

Nuocytes, identified as ILC2s, represent a new innate effector leukocyte that mediates Type 2 immunity, and produce IL-5 and IL-13 in response to IL-25 and IL-33.^4^ The cells have been described as a critical element for initiating Type 2 inflammation during *N. brasiliensis* infection.^4^ In addition, this cell type has also been shown to induce airway hyperreactivity in the absence of IL-13-producing Th2 cells in allergic lung inflammation.^5^ However, the contribution of nuocytes in AR is currently not well defined. Studies indicated that numbers of ILC2s were increased in the polyps from CRSwNP patients compared with nasal mucosa from CRSsNP patients or healthy controls.^19^ That is to say, ILC2s play a role in the upper airway allergic diseases. However, studies have demonstrated that ILC2s alone cannot lead to the symptoms of AR including tissue eosinophilia, which can be induced solely by activating ILC2s in lungs.^20^

One of our previous studies has reported that allergic responses can be exacerbated by OVA-induced nuocytes in a murine model of AR.^6^ In this current research, we administered intraperitoneally mouse rmIL-25 and rmIL-33 into wild-type and *Il17br^−/−^Il1rl1^−/−^*mice, and sorted and purified nuocytes from the mouse NALT and mLN. We found that there was no growth of nuocytes in the NALT of these two type mice after rmIL-25 and rmIL-33 applications. However, an elegant study showed proliferation of these cells in lung with rmIL-25 or rmIL-33 intranasal administration,^5^ which illustrates different significances of local and systemic immunity. The results in our study indicated expanding of nuocytes in the mLN of wild-type mice, and no growth in *Il17br^−/−^Il1rl1^−/−^*mice, which declared the indispensable role of IL-25 and IL-33 on the generation and activation of nuocytes, just as other studies have shown.^4^

According to our data, mLN-derived nuocytes from wild-type mice expressed more IL-13 compared to the cells from *Il17br^−/−^Il1rl1^−/−^*mice, and wild-type cells were induced to secrete more IL-5 and IL-13 by the mouse rmIL-25 and rmIL-33 in the culture. However, concentrations of IL-5 and IL-13 proteins and mRNAs were not increased in *Il17br^−/−^Il1rl1^−/−^* nuocytes after the two cytokines treatment. These findings suggest that combined *Il17br^−/−^Il1rl1^−/−^* knockout in mice completely deprives nuocytes of responses to IL-25 and IL-33.

We also found that the adoptive transfer of mLN-derived nuocytes from wild-type mice into the murine AR model exacerbated allergic condition, just like another study from us.^6^ Numbers of sneezing, nasal rubbing and invading eosinophils, and levels of IL-5, IL-13, IL-25 and IL-33 in the NLF were all enhanced significantly when compared to those of the AR mice. However, the above parameters were unchanged statistically when *Il17br^−/−^Il1rl1^−/−^* nuocytes were transferred to AR models. The outcomes reveal that nuocytes aggravate allergic state in AR mice models through the enhanced expressions of IL-5 and IL-13 by IL-25 and IL-33 produced from nasal mucosa epithelium,^10^ and the further upregulated productions of IL-25 and IL-33 by epithelial cells after the stimulation of nuocytes. As for the relationships between nuocytes and nasal epithelium, relevant additional studies should be performed.

To evaluate the influences of nuocytes on etiological mechanisms of AR, we determined whether allergen could establish AR models in *Il17br^−/−^Il1rl1^−/−^* mice through OVA and Alum according to the published procedures.^8^ Notably, we found there were no significant differences whether in numbers of sneezes, nasal rubs and infiltrating eosinophils, or in levels of Type 2 cytokines in the NLF between unstimulated and stimulated *Il17br^−/−^Il1rl1^−/−^* mice with OVA. However, after the adoptive transfer of wild-type nuocytes into the *Il17br^−/−^Il1rl1^−/−^* double-deficient mice, above parameters were upregulated statistically. As a result, AR models were settled successfully, and the allergic condition in nasal mucosa was regained. The data suggest that ILC2s like nuocytes and innate Type 2 cytokines such as IL-25 and IL-33 are absolutely necessary for the initiating of allergic state in a mouse model of AR.

## Conclusion

In conclusion, the study showed that IL-25 and IL-33 intraperitoneal administration contributed to expanding of nuocytes in the mLN of wild-type mice. mLN-derived wild-type nuocytes were induced to produce IL-5 and IL-13 by IL-25 and IL-33 in vitro, and aggravated allergic responses in AR mice in vivo. These findings indicate that nuocytes might play a proinflammatory role in the early stage of murine AR model, and the cells may become a new potential target in the future AR therapy.

## Funding

This work was supported by the 10.13039/501100001809National Natural Science Foundation of China (grant nº 81371076), the Shanghai Suburb Tertiary Hospital Clinical Capacity Building Project (grant nº SHDC12015905), and the Initial Funding of Huashan Hospital North of Fudan University (grant nº 2015102).

## Conflicts of interest

The authors declare no conflicts of interest.
